# Genome-Wide Identification of *GLK* Family Genes in *Phoebe bournei* and Their Transcriptional Analysis Under Abiotic Stresses

**DOI:** 10.3390/ijms26062387

**Published:** 2025-03-07

**Authors:** Yiran Lian, Liang Peng, Xinying Shi, Qiumian Zheng, Dunjin Fan, Zhiyi Feng, Xiaomin Liu, Huanhuan Ma, Shijiang Cao, Weiyin Chang

**Affiliations:** 1 College of Life Sciences, Fujian Agriculture and Forestry University, Fuzhou 350002, China; 18358678977@163.com (Y.L.); 13163835793@163.com (X.S.); 2 College of Forestry, Fujian Agriculture and Forestry University, Fuzhou 350002, China; pl3225320175@163.com (L.P.); qiumianzheng@163.com (Q.Z.); fandunjin@foxmail.com (D.F.); 3 Laboratory of Virtual Teaching and Research on Forest Therapy Speciality of Taiwan Strait, Fujian Agriculture and Forestry University, Fuzhou 350002, China; 4 College of Resources and Environment, Fujian Agriculture and Forestry University, Fuzhou 350002, China; savannah11210802@163.com; 5 State Key Laboratory of Tree Genetics and Breeding, College of Biological Sciences and Technology, Beijing Forestry University, Beijing 100083, China; liuxiaomin@bjfu.edu.cn (X.L.); mhh00928@163.com (H.M.)

**Keywords:** *P. bournei*, *GLK*, gene family, abiotic stress, chlorophyll

## Abstract

GOLDEN2-LIKE (GLK) transcription factors are crucial regulators of chloroplast development and stress responses in plants. In this study, we investigated the *GLK* gene family in *Phoebe bournei* (Hemsl.) Yen C. Yang, a near-threatened species important for forestry and wood utilization in China. We identified 61 *PbGLK* genes which were classified into seven subfamilies. Our analyses of their phylogenetic relationships, gene structures, and chromosomal distribution revealed diverse characteristics. Expression profiling under different tissues and abiotic stresses showed that *PbGLK25* and *PbGLK30* were particularly responsive to drought, heat, light, and shade stresses, with significant upregulation. These findings highlight the potential role of *PbGLK* genes in stress adaptation and provide insights for the genetic improvement of *P. bournei*.

## 1. Introduction

Transcription factors (TFs), also known as trans-acting factors, are proteins that bind to cis-acting elements within the promoter regions of target genes to regulate their expression through activation or inhibition [1,2]. These proteins control gene expression by attaching to specific cis-regulatory sequences within the promoters of target genes [3]. In the plant genome, approximately seven percent of coding sequences belong to TFs [4], underscoring their prevalence and importance in genomic regulation. TFs are a highly diverse group of regulatory proteins that play critical roles in various biological processes. For instance, they regulate plant responses to abiotic stresses such as saline–alkaline environments, temperature changes, drought, UV radiation, and other challenging conditions [5,6,7]. By modulating gene expression, TFs intricately control cellular processes and enable plants to respond effectively to external stimuli [8]. Additionally, TFs facilitate the optimization of the trade-off between stress tolerance and growth in plants, thereby enhancing their resilience and adaptability to adverse environmental conditions [9,10]. Therefore, understanding the factors and mechanisms that influence the functionality of plant TFs is crucial for deciphering the complex regulatory networks governing plant development, growth, and environmental adaptation [1,11].

Throughout a plant’s lifetime, it frequently encounters unfavorable or stressful conditions, including abiotic stresses such as drought, cold, salinity, and nutrient deficiency, as well as biotic stresses such as pathogen infections and herbivore attacks [12]. The GARP (Golden2, ARR-B, and Psr1) superfamily comprises a group of transcription factors that play essential roles in various biological processes, including hormonal signaling, nutrient sensing, chloroplast biogenesis, plant development, circadian clock oscillation, and floral transition [13,14]. Among these, GLK (Golden2-Like) proteins are key members of the GARP superfamily [13,15] and typically contain two conserved domains: a DNA-binding Myb-related domain and a dimerizing GCT domain [16]. Numerous studies have investigated their functions in plant growth, development, and resistance to abiotic stresses. The *GLK* gene in cotton has been shown to be significantly responsive to drought, salt, temperature stress, and developmental processes. Overexpression of the *GLK* gene in cotton significantly improves its resistance to these stresses, resulting in higher survival and yield under adverse conditions. These genes play a crucial role in regulating plant growth and stress tolerance by regulating cell responses to environmental stress sources [17]. In *Glycine max* (L.) Merr., research on *GLK* genes has revealed their critical roles in crop development and metal ion stress [18]. Additionally, studies on UV-B stress have identified that GLK transcription factors are highly conserved during evolution and may be associated with abiotic stress responses [19]. To cope with and resist these adverse conditions, plants have evolved multiple specific regulatory networks. For example, studies have identified candidate upstream regulators that enhance *GLK1*’s adaptability to varying environmental conditions by modulating its baseline activity, thereby revealing changes in the activity of the *GLK1*-centered regulatory network [20]. This highlights the critical role of GLK1 within these networks, as it belongs to the GLK transcription factor family, a significant group of transcription factors in plants that orchestrates responses to environmental stressors.

The *GLK* transcription factor family represents a significant group of transcription factors in plants [21]. Notably, research indicates that certain components of the *GLK* regulatory network have independently evolved in diverse species [22]. Some studies have found that while the function of *GLKs* is conserved across species, their binding sites are predominantly species-specific, with conserved binding sites typically located near photosynthetic genes regulated by *GLK* expression and whose expression is highly sensitive to *GLK* mutations [23,24]. This suggests a close association between *GLKs* and photosynthesis. Additionally, *GLK* transcription factors have been studied in various plants, including *Arabidopsis thaliana (L.) Heynh*, *Solanum lycopersicum* L. (tomato), *Oryza sativa* L. (rice), *Zea mays* L. (maize), *Hordeum vulgare* L. (barley), *Phyllostachys edulis* (Carrière) J. Houzeau (Moso Bamboo), and *Nicotiana tabacum* L. [25,26,27,28,29,30,31]. Many studies have highlighted the critical role of *GLKs* in chloroplast development. For example, in *A. thaliana*, GLKs directly bind to the promoter regions of PhANGs, thereby promoting chlorophyll biosynthesis, the assembly of the photosynthetic apparatus, and, subsequently, chloroplast development [32]. In tomatoes, BPG4 regulates chloroplast development and homeostasis by repressing *GLK* transcription factors and is involved in light and brassinosteroid signaling [26].

Few transcription factors have been identified to positively regulate chloroplast biogenesis, with the *GLK* gene family being an exception. Research indicates that members of the *GLK* family are essential for the formation and development of chloroplasts, thereby impacting plant photosynthesis and fruit quality [33,34,35]. It has been reported that GLK family transcription factors play a critical role in regulating chloroplast development and chlorophyll accumulation in the monocot maize and the eudicot *A. thaliana*, as well as in other plants, to adapt to varying environmental and developmental conditions [36,37,38]. Studies have shown that *GLK* genes assist in the coordinated regulation and synchronization of a certain set of nuclear photosynthesis genes, thereby optimizing photosynthetic capacity under various environmental and developmental conditions [24,39]. This enhanced photosynthetic efficiency subsequently promotes plant growth and development. In this study, an enrichment analysis of the *GLK* gene found that protein–chromophore linkage, photosynthesis, light harvesting, and the chlorophyll biosynthetic process were the most enriched biological processes. These results suggest that the *GLK* gene promotes chlorophyll biosynthesis and the expression of nuclear genes involved in the assembly of Lhcb (an important component of photosystem I (PSI) and photosystem II (PSII)) [39]. Although the important role of *GLK* genes in photosynthesis is well recognized, the vast majority of its biological functions remain unexplored and need further study.

In China, *P. bournei* is not only utilized as a preservative wood in wood art, shipbuilding, and architectural projects but is also an important tree species for use in artificial forestation [40,41]. However, its extensive use and afforestation effects have led to a decrease in its population [42]. Therefore, *P. bournei* has been classified as a near-threatened species, and continuous conservation efforts are imperative to protect it. It is particularly important to investigate the effects of various factors affecting the growth and development of *P. bournei*, hence restoring the amount of *P. bournei* in wild forests [43]. It has been suggested that the rapid growth of *P. bournei* under shaded conditions might be related to the increased expression of genes like *GLK*, which allows the plant to maintain high photosynthetic rates even in low-light environments [44]. However, the specific relationship between *GLK* genes and the growth of *P. bournei* remains to be further explored. In this study, we conducted a comprehensive study of the *GLK* gene in *P. bournei*, including genome-wide identification, phylogenetic analysis, gene structure, chromosome location, replication events, *cis*-acting element analysis, and expression levels under different tissues and stresses. It is anticipated that the findings from this study will not only enhance our current understanding of *GLK* genes in *P. bournei* and broader woody plant species but also provide a set of gene tools for their genetic improvement.

## 2. Results

### 2.1. Identification of PbGLK Genes in P. bournei

We identified a total of 61 *GLK* genes in *P*. *bournei* and renamed *PbGLK01* to *PbGLK61* (Table 1). The 22 GLK proteins encode amino acids ranging from 153aa to 941aa, with an average length of 404aa. The size of a protein is usually proportional to the length of its amino acid sequence. In the present study, the associated molecular weights of the 61 PbGLK proteins ranged from 17.56 kDa (PbGLK42) to 103.96 kDa (PbGLK48), with an average of 44.823 kDa, indicating a significant difference in the size of the GLK transcription factor proteins. A total of 34 PbGLK proteins were acidic (pI < 7.0), and the remaining 27 were basic, with a total value between 5.10 and 10.21. Meanwhile, six proteins (PbGLK7, PbGLK18, PbGLK23, PbGLK29, PbGLK42, and PbGLK52) were stable proteins, and the rest were unstable proteins (Instability Index > 40), ranging from 41.06 to 78.03. The data show that the aliphatic index ranged from 54.37 to 88.87, with a mean value of 70.97, reflecting the high thermostability of most PbGLK proteins. In addition, the mean value of the hydrophilicity of the PbGLK proteins was negative, which meant that all the PbGLK proteins were hydrophilic. According to the prediction of subcellular localization, there are 56 PbGLK proteins localized in the nucleus, followed by two each in the chloroplast and cytosol, and PbGLK1 in the mitochondrion.

### 2.2. Phylogenetic Analysis and PbGLK’s Chromosomal Locations

**Figure 1:**
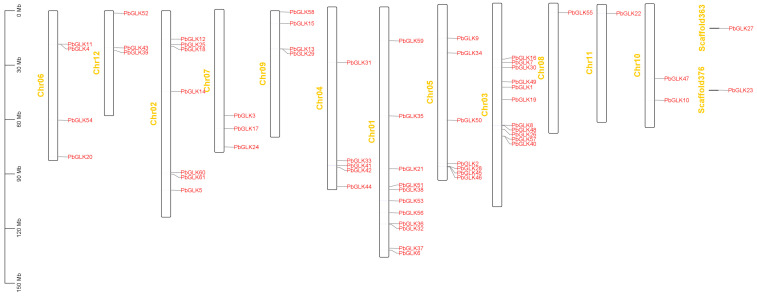
Distribution of *PbGLK* genes in *P. bournei* chromosomes. The chromosome number is shown on the left side of each chromosome. The scale on the left can be used to assess chromosomal length and gene position.

In this study, we determined that a total of 59 *PbGLKs* were distributed on 12 chromosomes (Chr), with *PbGLK23* and *PbGLK27* located in Scaffold376 and Scaffold363, respectively (Figure 1). We found that the distribution of *GLK* genes across the chromosomes was uneven. Most *PbGLK* genes were found in chromosomes 1 and 3, with both having 11 *PbGLKs,* whereas chromosomes 8 and 11 had the least *PbGLK* genes and carried only one *GLK* gene, named *PbGLK55*. Chr02, Chr03, Chr04, and Chr05 each had 3–5 *PbGLK* genes located in very close proximity.

**Figure 2:**
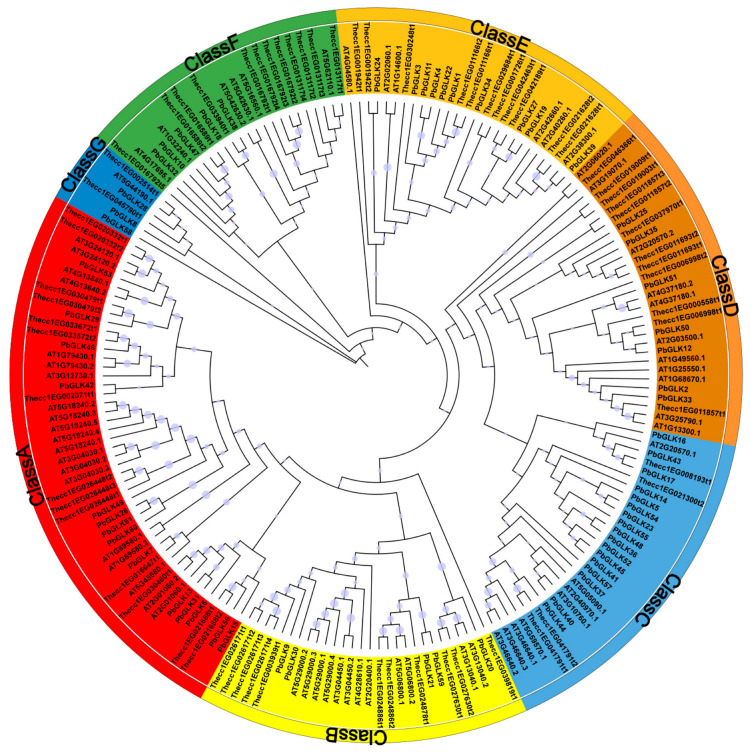
Phylogenetic tree of three plants’ GLK proteins. The different colored arcs represent different subfamilies of GLK proteins. The tree was built using 61 PbGLKs from *P. bournei*, 64 AtGLKs from *A. thaliana*, and 66 TcGLKs from *Theobroma cacao*. MEGA 11.0 was used to create a maximum likelihood phylogenetic tree, and the Bootstrap test replicate was set to 1000. All GLK proteins were divided into seven classes, with each class represented by a different color: Class A is represented by red, Class B is represented by yellow, Class C is represented by light blue, Class D is represented by dark brown, Class E is represented by light brown, Class F is represented by dark green, and Class G is represented by dark blue.

To investigate the evolutionary characteristics and relationships among the GLK proteins, we constructed a phylogenetic tree (Figure 2) using 61 GLK proteins from *P. bournei* alongside 64 and 66 GLK proteins from Arabidopsis and cocoa (*Theobroma cacao* L.), respectively. A total of 191 GLK proteins were divided into seven subfamilies (A-G) (Figure 2). The phylogenetic analysis showed that 48 *GLK* family members were concentrated in subfamily A, while subfamily G had only seven. Among them, the subfamily with the most *PbGLK* family members was subfamily C, with 17 members, accounting for 27.87% of the total. The second was subfamily A, with 14. However, subfamily G had only three *PbGLKs* (*PbGLK8*, *PbGLK28*, and *PbGLK58*). In contrast, among the *AtGLK* family members, subfamily A had the most with 20, and subfamily G had the least with only one. In the *TcGLK* family, subfamily A had up to 14 members, whereas subfamily G had a minimum of one member. These results indicated that the number of *GLK* genes in each subfamily differed among species.

### 2.3. PbGLK Gene Structure, Conserved Motif, and Domain Analysis

**Figure 3:**
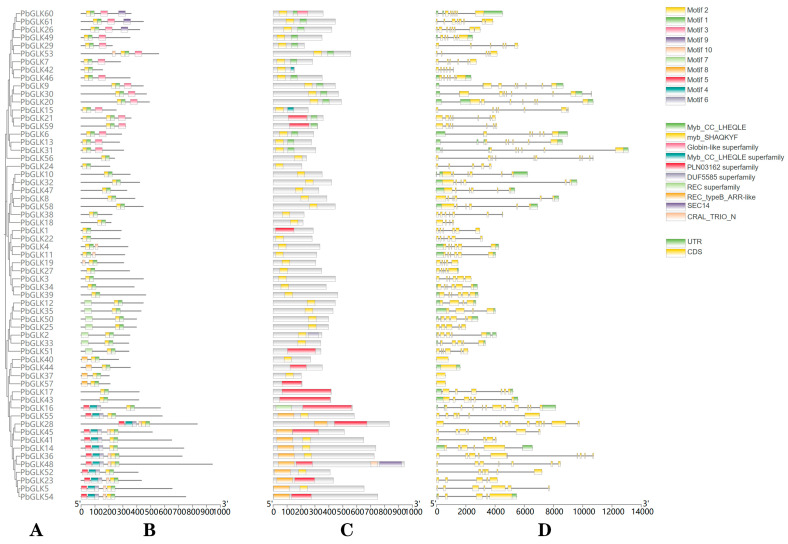
Protein motifs, domains, and structures of *GLK* gene family in *P. bournei.* (**A**) Class A is represented by red, Class B is represented by yellow, Class C is represented by light blue, Class D is represented by dark brown, Class E is represented by light brown, Class F is represented by dark green, and Class G is represented by dark blue. Phylogenetic tree constructed in MEGA using Maximum Likelihood algorithm using Bootstrap with 1000 replications. (**B**) Protein motifs in PbGLK members. The colorful boxes delineate different motifs. (**C**) Analysis of functional conserved domains was performed in the Pfam database (http://pfam.janelia.org/). (**D**) Gene structures of *PbGLK* gene family. CDS and UTR displayed using yellow and green rectangles, respectively. Black lines denote introns.

We analyzed the exon/intron structure of 61 *PbGLK* genes and found that the exon number of *GLK* genes ranged from one (*PbGLK37*, *PbGLK40*, *PbGLK44*, and *PbGLK57*) to 11 (*PbGLK16*) (Figure 3D). Subfamily G had the highest average exons (seven) per gene, and subfamily C had the lowest (five). There are also differences in gene structure between different subfamilies. For example, the genes in subfamily B have seven exons, while those in subfamily D have four to six exons.

Ten conserved motifs (Motif 1–10) of 61 PbGLK proteins were analyzed. Similar conserved motif compositions are often present in most GLK proteins from the same subfamily. Motif 1 and Motif 2 are present in all 61 PbGLK proteins, while several motifs appear only in specific subfamilies. For instance, only three out of 61 PbGLK proteins contain Motif 9, and all belong to subfamily A. All proteins of subfamily D contain Motif 7, which is present only in this subfamily. All members of subfamily B have Motif 3, and two (*PbGLK42*, *PbGLK56*) of the 14 genes of subfamily A have no Motif 3. In addition, the myb_SHAQKYF conserved domain can be identified in almost every *PbGLK* gene. It can be inferred from the analysis that the Myb_CC_LHEQLE domain is identified in both subfamilies A and B, and the expression levels of these two subfamilies are relatively high in different plant tissues (see figure in Section 2.7). Thus the biological role of proteins with the Myb_CC_LHEQLE domain can be further analyzed.

Therefore, the classification of *PbGLK* subfamilies was further supported by the differences in gene structure, conserved motif arrangement, and phylogenetic trees between the same subfamily and different subfamilies mentioned above. The diversity of the number, arrangement, and distribution of different motifs in the different subfamilies may be what differentiates them from one another.

### 2.4. Cis-Acting Elements: Analysis of the PbGLK Gene Family

**Figure 4:**
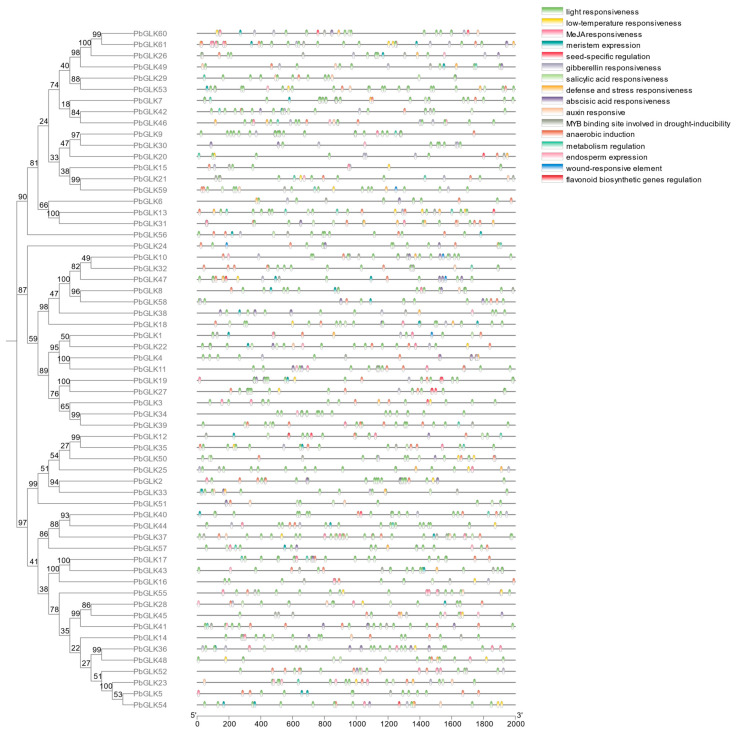
Schematic diagram of *cis*-element locations. The *cis*-element prediction of 61 *PbGLK* gene promoter sequences (−2000 bp) was analyzed using PlantCARE (https://bioinformatics.psb.ugent.be/webtools/plantcare/html/, accessed on 10 October 2024).

We identified *cis*-acting elements as DNA sequences located in the upstream promoter region of the genes, which can bind transcription factors to regulate gene function [45]. For example, it has been found that the HD-Zip transcription factor ArHDZ22 may regulate growth-related genes through its interaction with the hdzb *cis*-acting elements [46]. Additionally, experimental studies have identified a 150-bp *cis*-acting element within the promoter region of the *AtNRT2*.*1* gene, which plays a role in regulating gene expression in response to nitrogen and carbon states in plants [47]. Beyond natural promoter regions, transposable elements (TEs) can also influence gene expression by introducing new cis-acting regulatory elements into unconnected genomic locations [48]. Collectively, these findings highlight the critical role of *cis*-acting elements in determining the level and specificity of gene expression, a concept that has gained increasing recognition in recent years [49]. By detecting the 2000 bp promoter sequence of the upstream *PbGLK* genes, it was found that there were 16 *cis*-acting elements in the promoter region of the *PbGLK* gene family, involving four growth and development response elements, seven stress response elements, and five hormone response elements (Figure 4). Among them, the largest number of functional elements was light responsiveness, with 688 (48.18%). *Cis*-elements related to light-response functions are widely distributed in the *PbGLK* gene promoter, suggesting that the *GLK* gene may play a role in plant light responses. In addition to the most light-responsive components, a total of 500 components related to hormone response were found, including responses to various hormones such as auxin, gibberellin (GA), salicylic acid (SA), and methyl jasmonate (MeJA), indicating that the *PbGLK* gene holds significant importance in hormone response. Relatively few components include but are not limited to wound-responsive elements (5), seed-specific regulation (13), and endosperm expression (21). While the high abundance of light-responsive elements suggests that *PbGLK* family genes may be particularly sensitive to light, the presence of these elements alone does not confirm a definitive role in light responses. Additionally, the significant number of hormone-responsive elements indicates that *PbGLK* genes may also play important roles in hormone signaling pathways. Further functional studies are required to elucidate the specific roles of *PbGLK* genes in these processes.

### 2.5. Intraspecific Collinearity Analysis of PbGLK Genes

**Figure 5:**
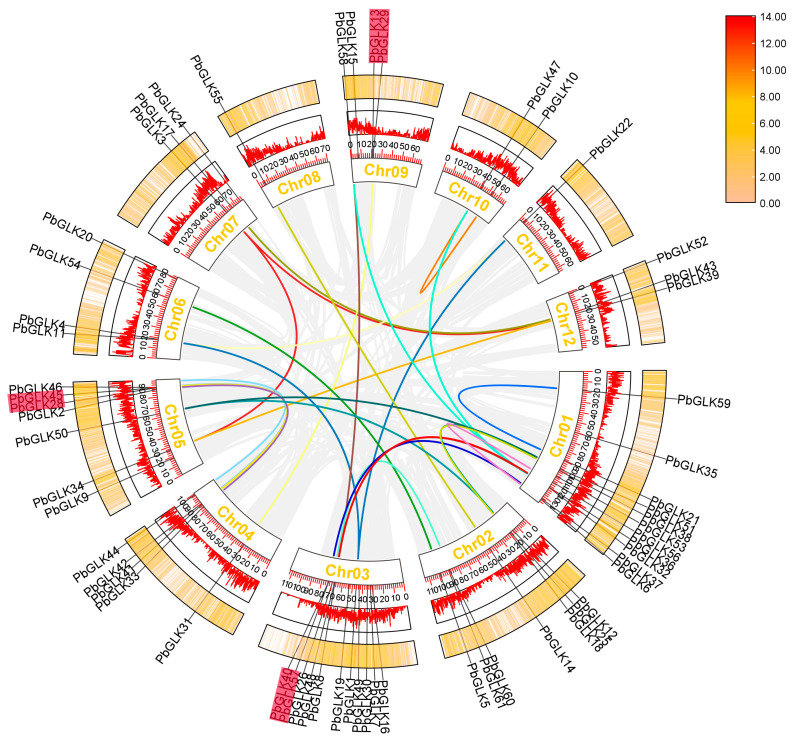
Analysis of inter- and intra-chromosomal fragment duplication of *GLK* genes in the *P*. *bournei* genome. The gray lines represent all synthetic blocks, and the colored lines specifically indicate the duplicated pairs among the 61 *PbGLK* genes. Tandem duplicated genes are set off by a red background.

Gene duplication events, including tandem repeat events and segmental duplication, play an important role in gene amplification and the generation of new functions, which are conducive to the development of species [50]. To better explore the evolutionary conservation of *GLK* genes in *P. bournei*, we examined genome-wide duplication events of *PbGLK* members in *P. bournei*. The results show a pattern of collinearity between duplicated gene pairs throughout the *P. bournei* genome (Figure 5). We found that there were 26 pairs of segmental duplications in the *P. bournei* genome, and the segment duplication events are mainly concentrated between Chr01 and Chr03 and between Chr04 and Chr05, which suggests that these genes may have arisen through gene duplication events, and that duplication events are the main drivers of the evolution of new functions for *PbGLK* genes.

### 2.6. Synteny Analysis Among PbGLK Genes

**Figure 6:**
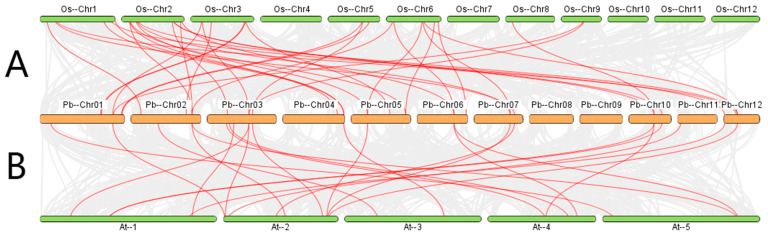
Synteny analysis of the genomes in *P. bournei*, *Oryza sativa* (**A**), and *A. thaliana* (**B**). Gray lines indicate collinear blocks within *P. bournei* and other plant genomes; the red line indicates collinear gene pairs.

To explore the possible evolutionary patterns of the *GLK* gene family of *P. bournei* in different plant varieties, we performed synteny analyses of the *GLK* genes of *P. bournei* with *GLK* genes of two other typical plants (Figure 6), including one typical dicotyledonous plant, *A. thaliana*, and one representative monocotyledonous plant, *Oryza sativa*. Our analysis shows that 22 *PbGLK* genes were collinear with *Oryza sativa*, and 19 *PbGLK* genes were collinear with *A. thaliana*. There were 34 pairs of *P. bournei* genes collinear with rice and 23 pairs of *P. bournei* genes collinear with *A. thaliana*. Although our synteny analysis revealed that more *PbGLK* genes were collinear with *Oryza sativa* than with *A. thaliana*, the level of collinearity does not directly equate to phylogenetic relationships or gene evolutionary distance. The observed patterns may be influenced by multiple factors, including genome rearrangement, gene loss, and duplication events [51]. Therefore, while these results suggest a higher degree of conservation between *PbGLK* genes and *Oryza sativa* genes compared to those in *A. thaliana*, further studies are needed to elucidate the precise evolutionary relationships among these genes.

The results showed that the *GLK* gene of *Oryza sativa* was genetically closer to the *GLK* gene of *P. bournei* than that of *A. thaliana*.

The proportion of *P. bournei* and other species containing similar *GLK* genes suggests, to some extent, the evolutionary relationship of *GLK* genes among *P. bournei*, monocots, and dicots.

### 2.7. Expression Analysis of PbGLKs in P. bournei Tissues

**Figure 7:**
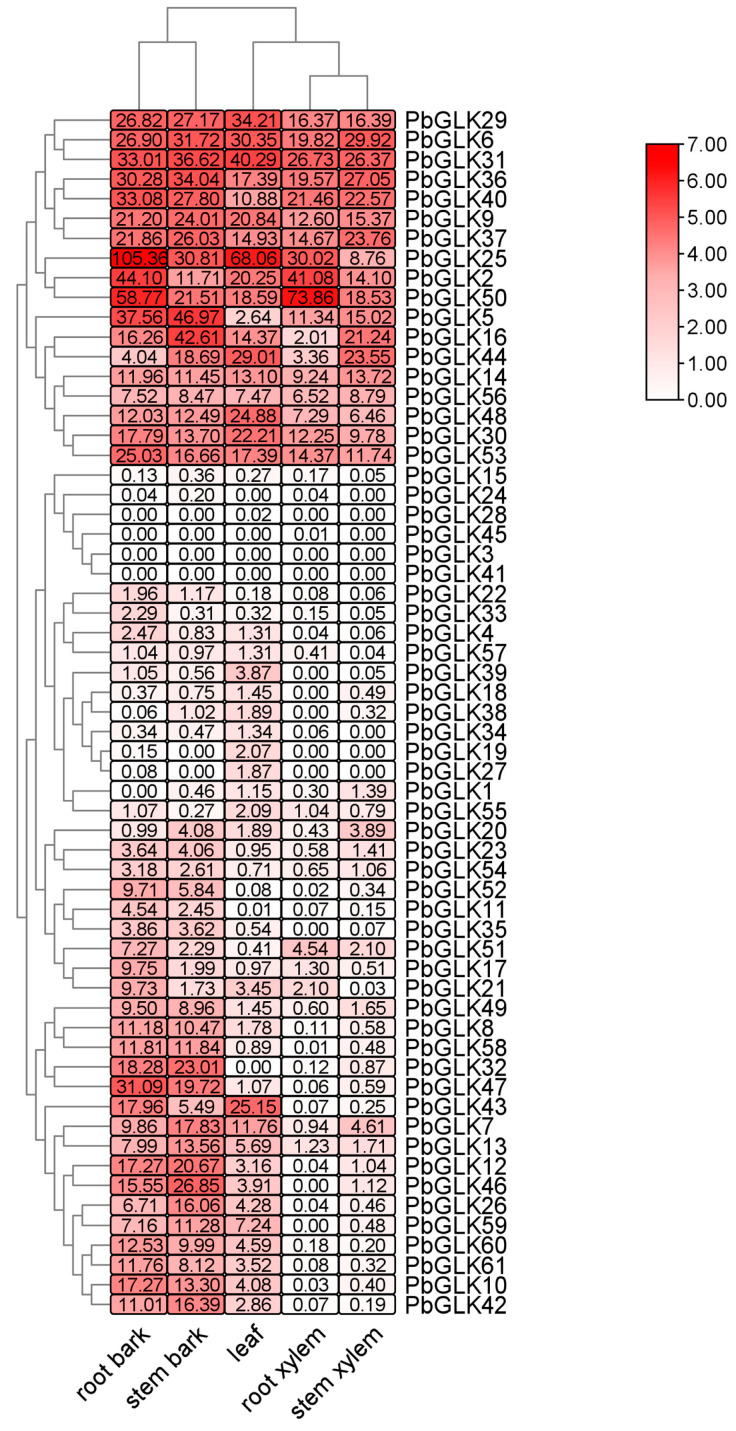
Expression profiles of 61 *PbGLK* genes across various tissues depicted in a schematic diagram, where red blocks indicate high expression levels and white blocks signify low expression levels.

We compared the expression levels of 61 *PbGLKs* across five tissues of *P*. *bournei*: root bark, stem bark, root xylem, stem xylem, and leaf (Figure 7). Apart from a few specific genes, most genes were generally expressed across the five selected plant tissues. There are only three genes in subfamily G, namely, *PbGLK8*, *PbGLK28*, and *PbGLK58*, among which *PbGLK8* and *PbGLK58* are expressed in all tissues and have the highest expression levels in root bark and stem bark, while *PbGLK28* is only slightly expressed in leaves. In subfamily F, *PbGLK32* was not expressed in leaves, *PbGLK18* and *PbGLK38* were not expressed in root xylem, and the other two genes were expressed in all tissues and were highly expressed in the epidermis. The average gene expression of subfamily E was the lowest among the seven subfamilies. In contrast to subfamily E, subfamily D had the highest average gene expression. *PbGLK25* exhibited extremely high gene expression in the root bark and leaves, while *PbGLK50* showed extremely high gene expression in the root bark and xylem. It is worth noting that the expression of *PbGLK25* was the highest among *61 PbGLKs*. Most genes in subfamilies A, B, and C were expressed in all tissues, and their expression levels were relatively high. These findings suggest that the expression levels of *PbGLK* vary within and between the subfamilies.

Overall, the expression levels were lowest in the xylem, whereas the epidermis showed higher expression compared to both the xylem and leaves, with the root epidermis exhibiting the highest expression levels. This may be due to the fact that *PbGLK* genes are largely involved in epidermal growth and development.

### 2.8. Expression of PbGLK Genes Under Abiotic Stress

**Figure 8:**
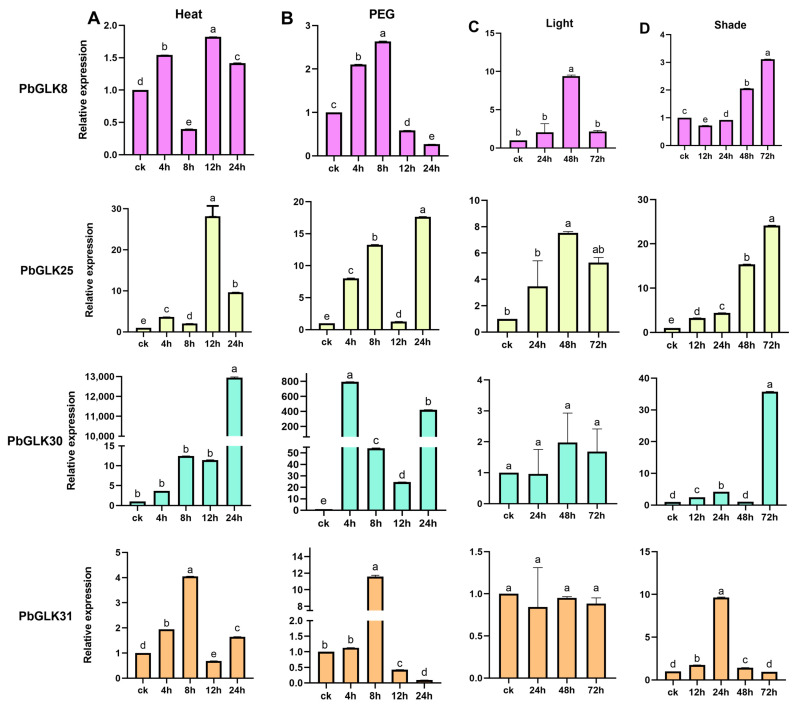

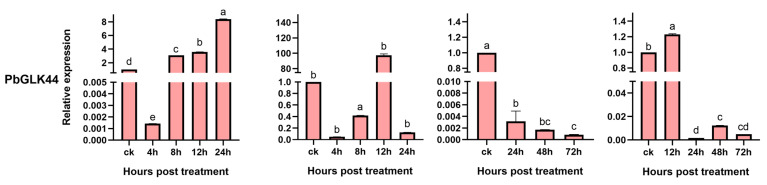
The expression profiles of *PbGLK* genes in *P*. *bournei* were detected via qRT-PCR in response to drought, heat, light, and shade stress. (**A**) Relative gene expression levels under heat stress over the same time period (0, 4, 8, 12, and 24 h). (**B**) Relative gene expression levels under drought treatment over the same time period (0, 4, 8, 12, and 24 h). (**C**) Relative gene expression levels under light stress (0, 24, 48, and 24 h). (**D**) Relative gene expression levels under shade stress (0, 12, 24, 48, and 72 h). Significant differences (*p* < 0.05) were determined by the LSD test, expressed by different letters above the bar. Different letters indicate significant differences between groups, while the same letters indicate no significant differences between groups.

Due to their immobility, plants are exposed to a more variable environment compared to other organisms, with abiotic stresses such as drought, heat, light, and shade stress significantly impacting their growth and development [52,53,54]. In order to study the role of *PbGLK* genes in plant responses to these stress, five *PbGLK* genes, *PbGLK8*, *PbGLK25*, *PbGLK30*, *PbGLK31*, and *PbGLK44*, were selected from seven subfamilies (A–G) for real-time quantitative polymerase chain reaction (qRT-PCR) based on the subfamily they belong to (less than two from one subfamily) (Figure 2), the number of *cis*-acting elements in each gene (Figure 4), and their expression levels in various tissues of *P. bournei* (Figure 8).

In response to elevated temperatures, the expression levels of various genes exhibited changes across distinct time intervals. Notably, following exposure to high temperatures, the expression levels of the genes *PbGLK25* at 12 h and *PbGLK44* at 24 h were observed to rise by approximately 28-fold and 8-fold, respectively, relative to their pre-treatment levels. Additionally, the expression of the *PbGLK30* gene was markedly elevated at the 24 h mark, leading us to speculate that these genes are associated with plant adaptation to heat stress. In arid environments, except *PbGLK25* and *PbGLK30*, the expression of most genes gradually decreases after 12 h. The expression level of *PbGLK30* reached its peak after 4 h, and the maximum expression level of *PbGLK30* was about 793 times that of the control group, indicating a potentially crucial role of the *PbGLK30* gene in the plant’s response to drought conditions. When the genes were exposed to light stress, most of the genes showed a trend of low–high–low change. Compared to the control group, the expression levels of the genes *PbGLK44* and *PbGLK31* were reduced, with *PbGLK44* showing the most pronounced suppression under light stress conditions. We therefore hypothesized that the *PbGLK44* gene may not be beneficial for plants in effectively managing light stress. When examining the impact of shade stress on gene expression, it becomes evident that the expression of *PbGLK44* and *PbGLK31* diminished most notably over time. Conversely, the expression of the other three genes continued to rise even until the 72 h mark. Among them, *PbGLK25* and *PbGLK30* exhibited the highest levels of expression, increasing 24-fold and 36-fold, respectively, compared to the control group. These findings suggest that *PbGLK25* and *PbGLK30* may play important roles in the plant response to shade stress.

To summarize, the expression levels of almost all genes reached their peaks during specific intervals under drought, heat, light, and shade stress conditions. The expression of the *PbGLK* genes increased across most treatment periods compared to the beginning, suggesting that *PbGLK* could be vital for plants in managing these four types of stress. Notably, *PbGLK25* and *PbGLK30* displayed pronounced responses to drought, heat, light, and shade stress, indicating that *P. bournei* may be primarily regulated by some specific *PbGLK* genes when facing these stresses. While these findings provide insights into the potential functions of PbGLK genes under different stress conditions, further experiments are necessary to validate their exact roles and functional significance.

## 3. Discussion

GLK transcription factors have been demonstrated to be crucial for chloroplast development [23,25,55], photosynthesis [27,56], stress resistance [30,57], and hormone responses [22,30]. Due to the importance of the *GLK* gene family in abiotic stress and plant growth and development, it has been studied in plants such as *A. thaliana* [25] and maize [58]. To date, the whole-genome analysis of *GLK* has been conducted in plants such as Moso Bamboo, *Gossypium hirsutum* L., and *Camellia sinensis* (L.) Kuntze (Camellia) [12,30,45], but a whole-genome analysis of *P. bournei* has still not been carried out. Therefore, the genome-wide characterization and expression analysis of the *GLK* gene family in *P. bournei* will enhance our understanding of *GLK* genes within the same plant and the *GLK* gene family across different species, providing deeper insights into the functions and roles of these genes.

In this study, we used bioinformatics to identify the *PbGLK* gene family and discovered 61 *PbGLK* genes separated into seven subfamilies (Figure 2). The *GLK* genes in Camellia and *Eucalyptus grandis* W. Hill ex Maiden were also divided into seven subfamilies for analysis [22,59]. In comparison, the number of *GLK* genes in the G/I subfamily of tea tree and *P. bournei* was the lowest, while those distributed in the A/VII subfamily of *Eucalyptus grandis* and *P. bournei* were relatively high, accounting for the second highest and highest, respectively, in their respective comparisons. In addition, we found that *P. bournei* had three and 13 fewer *GLK* members than *A. thaliana* and cocoa, respectively (Table S1). The *GLK* family members from the three groups were intermingled, with no distinct branches observed (Figure 2). These results suggest that the *GLK* gene family shows a similar evolutionary pattern in *P. bournei*, Camellia, and *Eucalyptus grandis*, with members distributed in multiple subfamilies. Additionally, the *GLK* gene family in dicots exhibits limited evolutionary divergence and remains highly conserved across species. However, the differential expansion or contraction of specific subfamilies (e.g., A/VII and G/I) among species may reflect lineage-specific adaptations to varying ecological niches and environmental pressures. For instance, the expansion of the A/VII subfamily in *P*. *bournei* and *Eucalyptus grandis* could be linked to their adaptation to woody growth habits and stress tolerance mechanisms, as these genes may play roles in regulating light-responsive pathways and enhancing photosynthetic efficiency under adverse conditions. Conversely, the reduced size of the G/I subfamily in P. bournei and tea tree might indicate functional redundancy or specialization within this group, potentially driven by niche-specific selective pressures. Although our data suggest that the *GLK* gene family is conserved to some extent in these species, to fully understand the evolutionary dynamics and conservation of the *GLK* gene family in dicotyledonous plants, further comprehensive analyses in a wider range of dicotyledonous species are needed in subsequent studies.

*GLK* often works synergistically with other genes or with proteins and substances that are the products of their expression. For example, the interaction between *GLK* and *TCP15* may help coordinate the expression of cell expansion genes and genes involved in the development of photosynthetic organs [55]; transgenic GLK facilitates tumor metastasis and cellular migration by leveraging the IQ motif within the scaffold protein that contains GTPase-activating protein 1 (IQGAP1) [60]; and BPG4 interacts with GLK, affecting the amount of chlorophyll and the size of the light collection complex [32]. In addition, AcGLK1 and AtGLK transcription factors are also thought to affect chloroplast development or chlorophyll accumulation [61,62,63]. Additionally, *GLK* is involved in modulating the various activities of chlorophyll synthases, including δ-aminoacetobulinate dehydratase (ALAD), porphyrinogen deaminase (PGBD), and protoporphyrinogen oxidase (PROTOX) [64]. These studies suggest that GLK often acts in concert with other factors and plays an important role in chloroplast development and accumulation. It is worth noting that our analysis of the subcellular localization prediction of *PbGLKs* showed that most *PbGLKs* were located in the nucleus. Only *PbGLK6* and *PbGLK21* were located in chloroplasts (Table 1), a finding similar to the results of a whole-genome analysis of *GLK* in *Eucalyptus grandis, Citrus* Cultivar Kanpei, and so on [12,65], again suggesting that the evolution of the *GLK* gene family is conservative. In addition, *PbGLK21* was highly expressed in the root epidermis, root xylem, stem epidermis, stem xylem, and leaves (Figure 7). We speculate that the function and action of this gene are related to the physiological activity of chloroplasts, which needs to be verified by further experiments.

As has been shown, gene duplication events are not only an important mechanism for gene diversification but also provide opportunities for the acquisition of new gene functions [62]. In this study, we analyzed variants in conserved motifs in the GLK protein family and discussed the role of these variants in the diversification of gene functions. The myb_SHAQKYF transcriptional repressor, found in *A. thaliana*, can regulate leaf wax biosynthesis through the transcriptional repression of DEWAX [64]. In our analysis, myb_SHAQKYF was identified as a key conserved structural domain of the *PbGLK* gene family in almost all *PbGLK* genes (Figure 3C). It is suggested that this structural domain may have an important regulatory role in gene function and that the *GLK* genes may be regulating non-stomatal transpiration and, thus, responding to stress by regulating leaf wax.

Gene duplication and conserved motif variation are bridges connecting different subfamilies [66]. In *P. bournei*, all *PbGLKs* contain Motif 1, which varies between subfamilies but is conserved in each subfamily. This conservatism may reflect the importance of specific genes in specific biological functions [45]. However, Motifs 17 and 18 were only observed in subfamilies C and D, suggesting that these specific motifs may be associated with subfamily-specific functions. In Moso Bamboo, Motif 5 is present in subfamilies 1, 2, 3, 4, 5, 6, and 7, while Motifs 5, 6, and 8 occur in subfamily 10. It can be inferred that the amino acid variation in Motif 5 may be an important cause of its phylogenetic differentiation [30]. In the conserved motif analysis of this experiment, we found that all members of subfamily D contain Motif 7, while none of the genes of subfamily C contain this motif, suggesting that the loss of Motif 7 may be related to the evolutionary division of the subfamily (Figure 3B). All members of subfamily B and some members of subfamily A may have arisen from tandem duplication gene events, whereas *PbGLK42* and *PbGLK56* do not contain Motif 3. This loss of Motif 3 may have led to the divergence of the *PbGLK* gene family through tandem gene duplication events. In this context, the different evolutionary pathways of *PbGLK42* and *PbGLK56* may represent the starting point for functional diversification. Duplication events not only promote the expansion of gene family size but may also trigger the loss or gain of specific motifs, which, in turn, leads to the diversification of gene functions.

Abiotic stresses such as drought, heat, light, and shade significantly impact plant growth and development [67], and our findings indicate that *GLK* genes may play crucial roles in plant responses to these stresses by integrating developmental and stress-responsive pathways. Chloroplasts are essential for photosynthesis and act as environmental sensors [68], but their function can be compromised under stress conditions [69]. *GLK* genes, which regulate chloroplast development, might help maintain chloroplast integrity during stress; for instance, the substantial upregulation of PbGLK30 under drought stress (793-fold) suggests its potential role in protecting chloroplast function and optimizing photosynthetic efficiency when water is scarce. Furthermore, the abundance of light-responsive cis-acting elements in the promoters of *GLK* genes highlights their involvement in balancing light signaling and stress adaptation. Under light stress, the suppression of PbGLK44 indicates a possible negative regulatory role in preventing excessive photosynthetic activity that could lead to oxidative damage, while the upregulation of PbGLK25 and PbGLK30 under shade stress reflects their importance in compensating for reduced light availability. Additionally, the presence of numerous hormone-responsive elements in the *PbGLK* gene promoters implies that these genes participate in hormone-mediated stress responses, potentially interacting with pathways involving auxin, gibberellin, salicylic acid, and methyl jasmonate. The distinct expression patterns of *PbGLK* genes across different stress conditions further demonstrate their functional diversity, with PbGLK25 and PbGLK30 showing pronounced responses to multiple stresses, suggesting they are key regulators in plant stress adaptation. While these insights provide a foundation for understanding the roles of *GLK* genes in abiotic stress responses, further functional studies are necessary to clarify the underlying mechanisms and validate their significance in plant resilience.

**Figure 9:**
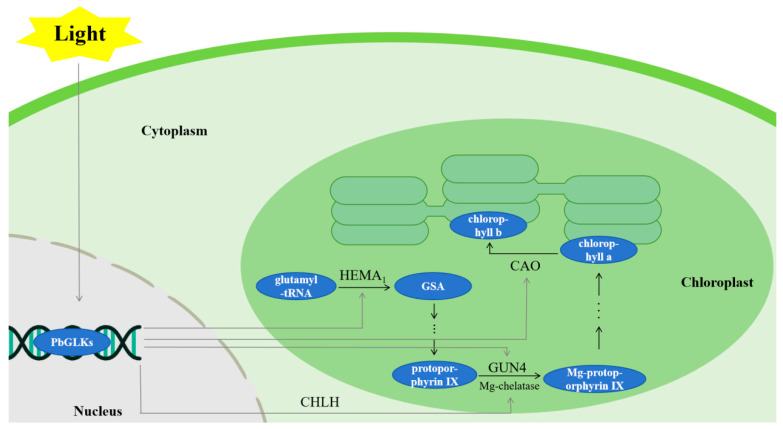
Prediction model of *GLK* pathway in chlorophyll biosynthesis of *P. bournei*. HEMA1 (glutamyl-tRNA reductase [GluTR]) initiates the crucial and rate-determining step in the biosynthesis of tetrapyrroles, CHLH (the H subunit of Mg-chelatase) plays a pivotal role in channeling tetrapyrrole molecules towards the chlorophyll production pathway, GUN4 is required for efficient Mg-chelatase activity, and CAO catalyzes the conversion of chlorophyllide a to chlorophyllide b.

Research has elucidated the impact of light and hormonal signals on chloroplast development and chlorophyll biosynthesis within tea tree (*Camellia sinensis*) [70]. The direct targeting of a common promoter cis-box (G-box) can optimize photosynthetic performance and growth through a series of processes [71], so we speculate that gene response to stress and the presence of *cis*-elements in plants jointly affect plant photosynthetic performance. In the above stress analysis, some *GLK* genes showed significant responses to light and shade, suggesting that these genes may be involved in responses to abiotic stresses (Figure 8). In addition, the above promoter analysis showed that, although *cis*-elements related to hormone, stress, and developmental regulation are also found in GLK promoters, those that respond to light are the most abundant. Under light conditions, the expression of *PbGLK* is closely related to four key enzymes of chlorophyll biosynthesis [72,73,74]. Based on the above findings, the role of *PbGLK* genes on chlorophyll synthesis under light conditions can be predicted (Figure 9).

Although this study provides some insights into the evolutionary patterns and functions of the *GLK* gene family in *P. bournei*, some limitations should be acknowledged. First, we did not perform subcellular localization experiments for the PbGLK proteins. This decision was based on the high conservation of *GLK* genes across species and consistent reports in the existing literature that indicate their nuclear localization. Given this strong evidence, we believe the predictions of subcellular localization are reliable. Second, while our data suggest a certain degree of conservation of the *GLK* gene family in these species (*P. bournei*, Camellia, and *Eucalyptus grandis*), a more comprehensive analysis of a broader range of dicotyledonous plants is required to fully understand the evolutionary dynamics and conservation of the *GLK* gene family in dicots. Additionally, although the presence of high-abundance, light-responsive, and hormone-responsive *cis*-acting elements implies potential roles of *PbGLK* genes in light- and hormone-signaling pathways, their exact functions remain unclear and require further experimental validation. The current study primarily relies on bioinformatics analyses, such as sequence comparisons and cis-element predictions, but lacks direct functional evidence from experiments like gene knockout, overexpression, or promoter activity assays. Finally, the roles of *PbGLK* genes in biological processes, including light and hormone signaling, need to be experimentally validated through functional studies to confirm their contributions to these pathways.

## 4. Materials and Methods

### 4.1. Genome Data and Plant Material Source

The genome sequence data and annotation information of *P. bournei* were downloaded from the Sequence Archive of China National GeneBank Database (CNSA) with accession number CNP0002030 [70]. Genome sequence files of *A. thaliana* and *O. sativa* were acquired from EnsemblPlents and Phytozome v13, respectively. The RNA-seq data from different tissues of *P. bournei* were downloaded from BioProject with accession number PRJNA628065. The plant materials were derived from one-year-old *P. bournei* seedlings cultured in an artificial climate box under different treatments. *P. bournei* seedlings with the same growth potential for one year were selected for treatment, and the materials were divided into the control group and stress treatment group, with 30 strains in the treatment group and three strains in the control group. Every two strains in the treatment group were used as a biological replicate, and three groups of biological replicates were set in each time period. During the stress treatment period, the seedlings were cultured in an artificial climate incubator with a temperature of 25 °C and a humidity of 75%. Then, the *P. bournei* seedlings were exposed to drought stress (10% PEG6000) and heat stress (40 °C). Samples of mature leaves were collected at 0 (CK), 4, 8, 12, and 24 h. Mature leaf samples under light stress (light 24 h/d) and shade stress (light 0 h/d) were picked at 0 (CK), 24, 48, and 72 h and 0 (CK), 12, 24, 48, and 72 h, respectively. The control group (CK) was treated with distilled water and normal growth conditions. After various treatments, *P. bournei* leaf samples were collected and stored in liquid nitrogen at −80 °C for RNA extraction.

### 4.2. Identification and Physical and Chemical Property Analysis

The conserved domain of the *A. thaliana GLK* gene family was downloaded from PlantTFDB. A local BLASTp search was used to compare the conserved domain between *P. bournei* and *A. thaliana* to screen the candidate *GLK* genes in *P. bournei* [71]. The repetitive results of the BLASTp search were removed. The identified protein sequences of *GLK* genes from *P. bournei* were submitted to the NCBI for BLASTp to perform further searches. To further identify *GLK* gene family members, the GLK conserved domain HMM model (PF00249) was downloaded from the Pfam database using HMMER-3.2.1 with an e-value <10^−5^ and other parameters by default. After the identification of *GLK* genes in *P. bournei*, the online website ExPASy was used to analyze the physical and chemical properties of the GLK proteins that were identified [72].

### 4.3. Chromosomal Distribution and Gene Duplication of PbGLK Genes

TBtools was used for grepping the location information of the *PbGLK* genes from the genome (FASTA) file and the annotation (GFF) file of *P. bournei* [72]. The syntenic relationships of *PbGLK* were determined using MCScanX (version 1.00) with default parameters and plotted using TBtools-II v2.105 [73].

### 4.4. Collinearity Analysis of PbGLK Genes

The syntenic relationships between *PbGLK* genes and *GLK* genes from *A. thaliana* and *O. sativa* were determined using MCScanX software. TBtools was used for visualization.

### 4.5. Phylogenetic Analysis

The sequences of GLK proteins of *P. bournei*, *A. thaliana*, and *O. sativa* were aligned using the Muscle program of MEGA11 with default settings for constructing maximum likelihood (Bootstrap replications: 1000) phylogenetic tree [74,75]. iTOL (https://itol.embl.de/, accessed on 13 November 2024) was used to improve and beautify the phylogenetic tree.

### 4.6. Analysis of Conserved Motifs and Gene Structures

The protein sequence of *P. bournei* was identified using the online software MEME (https://meme-suite.org/meme/tools/meme, accessed on 9 October 2024), and the predicted value of the motif number was 10. The multiple GLK protein sequence alignment was carried out using Jalview software (version 2_11_4_0). We used a batch CD-search with default parameters to detect the conserved domains of the PbGLK proteins.

### 4.7. Promoter Cis-Element Analysis of PbGLK Genes

To explore the *cis*-acting elements in the sequence, we extracted the upstream 2000 bp sequences from the *P. bournei* genome. The online database PlantCARE was used to identify and analyze the *cis*-acting regulatory elements in the promoter region of the *PbGLK* genes. After selection and categorization, the data were visualized using TBtools software.

### 4.8. RNA Extraction and qRT-PCR Analysis

Total RNA extraction was carried out using an RNA Extraction Kit (Omega Bio TEK, Shanghai, China) for both the control and the stress-treated samples. Following the instructions of manufacturers, EasyScript One-step gDNA Removal and cDNA Synthesis SuperMix (Transgen, Beijing, China) were utilized to synthesize the cDNA. qRT-PCR was subsequently performed using TransStart top green qPCR SuperMix (Transgen, Beijing, China). The specific primers used in the qRT-PCR experiment were designed by TBtools and are listed in Table S2. The mixture solution of the qRT-PCR reaction is composed of 1 L of cDNA, 2 L of specific primers, 10 L of SYBR Premix Ex TaqTM II, and 7 L of ddH_2_O. The qRT-PCR reaction process was as follows: pre-degeneration at 95 °C for 30 s, then 40 cycles of denaturation at 95 °C for 5 s, 60 °C for 30 s, 95 °C for 5 s, 60 °C for 60 s, and 50 °C for 30 s. The internal reference gene was PbEF1α (GenBank No. KX682032) [76]. The relative expression of *PbGLK* genes was calculated using the 2^−∆∆CT^ method, and one-way ANOVA and Duncan multiple comparison tests were performed using the GraphPad Prism9.0 software (https://www.graphpad.com/) [77]. One-way ANOVA was conducted to determine whether there were significant differences among the means of the groups. Subsequently, Duncan’s multiple comparison test was performed to identify which specific groups exhibited significant differences. This test was chosen because it is suitable for comparing multiple groups with unequal sample sizes while effectively controlling the Type I error rate. After determining the experimental groups, differences among groups were annotated using the ABCD annotations. This method was selected because it is more appropriate for representing significance when dealing with multiple time points. To ensure robustness, all quantitative PCRs were conducted with three biological repeats and three technical replicates.

## 5. Conclusions

In this comprehensive study, we conducted a genome-wide analysis of the *GLK* gene family in *P. bournei*, elucidating its phylogenetic relationships, gene structures, and expression under various abiotic stresses. Our findings have significant implications for understanding the role of *GLK* genes in plant growth, chloroplast development, and stress adaptation, particularly in the context of woody plants.

Firstly, we identified 61 *PbGLK* genes in *P. bournei*, which were classified into seven subfamilies based on phylogenetic analysis. This classification was supported by differences in gene structure, conserved motif arrangement, and chromosomal distribution. Notably, the C subfamily contained the largest number of members, indicating its potential importance in the functional diversification of *PbGLK* genes. The uneven distribution of *PbGLK* genes across 12 chromosomes, along with evidence of segmental duplication events, suggests that gene duplication has played a crucial role in the expansion and functional evolution of this gene family.

Our analysis of cis-acting elements in the promoter regions of PbGLK genes showed a high prevalence of both hormone-related and light-responsive elements, with a particularly significant occurrence of light-responsive elements. This suggests that these genes are likely involved in regulating chloroplast development and photosynthesis under varying light conditions. This finding is further supported by the expression analysis, which showed that specific *PbGLK* genes, such as *PbGLK25* and *PbGLK30*, exhibited significant upregulation under drought, heat, light, and shade stresses. These genes may serve as key regulators in enhancing stress tolerance and maintaining photosynthetic efficiency in *P. bournei*.

Although the implication of our findings is for fundamental research, given the near-threatened status of *P. bournei* and its ecological and economic importance in China, the identification of stress-responsive *PbGLK* genes provides a valuable genetic resource for the genetic improvement of this species. For instance, the overexpression of *PbGLK25* and *PbGLK30* could potentially be explored in breeding programs aimed at developing *P. bournei* varieties with enhanced tolerance to abiotic stresses, thereby contributing to forest conservation and sustainable forestry practices.

Moreover, our study highlights the conserved and divergent roles of *GLK* genes across different plant species. The synteny analysis with *A. thaliana* and *O. sativa* revealed both conserved and species-specific patterns, suggesting that the *GLK* gene family has undergone lineage-specific evolution while retaining core functions related to chloroplast development. This insight could guide comparative studies on *GLK* genes in other woody plants and crops, facilitating the identification of key regulatory genes involved in stress adaptation and growth optimization.

In conclusion, this study enhances our understanding of the *GLK* gene family in *P. bournei* and provides a foundation for future research on the molecular mechanisms underlying their functions. The identified stress-responsive *PbGLK* genes offer promising targets for genetic engineering and breeding efforts aimed at improving the resilience of *P. bournei* and other related species. Future work could focus on functional validation of these genes through transgenic approaches, as well as exploring their interactions with other regulatory networks involved in stress responses and chloroplast biogenesis.

## Figures and Tables

**Table 1:** Detailed information on 61 *PbGLK* genes of *P. bournei* and their encoded proteins.

Gene Accession	Gene Id	Size/aa ^1^	MW ^2^/Da	Theoretical pI ^3^	Instability Index	Aliphatic Index	GRAVY ^4^	Subcellular Localization
OF25727-RA	*PbGLK1*	287	32,398.86	8.34	51.17	80.17	−0.559	Mitochondrion
OF02278-RA	*PbGLK2*	349	39,145.71	6.66	78.03	57.02	−0.98	Nucleus
OF27064-RA	*PbGLK3*	446	50,230.21	5.27	52.6	73.52	−0.682	Nucleus
OF17914-RA	*PbGLK4*	335	38,287.71	8.28	70.16	70.42	−0.796	Nucleus
OF08332-RA	*PbGLK5*	652	70,642.4	5.9	46.14	78.97	−0.452	Nucleus
OF28298-RA	*PbGLK6*	290	31,657.62	6.55	45.16	77.34	−0.644	Chloroplast
OF09746-RA	*PbGLK7*	282	31,675.63	8.59	33.76	72.98	−0.827	Nucleus
OF23687-RA	*PbGLK8*	385	43,157.17	6.67	58.11	57.77	−0.671	Nucleus
OF01303-RA	*PbGLK9*	445	49,256.73	5.14	65.21	65.73	−0.716	Nucleus
OF20594-RA	*PbGLK10*	351	39,354.17	8.62	50.29	69.97	−0.599	Nucleus
OF17917-RA	*PbGLK11*	312	35,624.58	6.31	65.28	66.25	−0.877	Nucleus
OF04301-RA	*PbGLK12*	445	48,861.35	6.26	60.64	59.28	−0.807	Nucleus
OF03170-RA	*PbGLK13*	275	30,294.98	9.1	51.95	73.75	−0.757	Nucleus
OF08790-RA	*PbGLK14*	736	79,576.63	6.16	43.31	79.47	−0.43	Nucleus
OF16131-RA	*PbGLK15*	251	28,606.27	8.7	54.3	72.31	−0.747	Nucleus
OF09825-RA	*PbGLK16*	568	62,996.06	5.98	52.61	72.29	−0.571	Nucleus
OF24628-RA	*PbGLK17*	416	45,705.35	5.89	48.05	72.88	−0.576	Nucleus
OF04095-RA	*PbGLK18*	214	24,016.97	8.59	33.18	72.99	−0.636	Nucleus
OF24353-RA	*PbGLK19*	305	34,766.86	8.88	63.42	62	−1.178	Nucleus
OF26355-RA	*PbGLK20*	489	53,954.06	6.33	67.79	71.25	−0.722	Nucleus
OF02538-RA	*PbGLK21*	358	39,814.67	6.19	61.43	64.78	−0.637	Chloroplast
OF14101-RA	*PbGLK22*	279	32,386.57	6.32	54.62	72.01	−0.706	Nucleus
OF08001-RA	*PbGLK23*	432	47,244.12	6.06	38.97	83.47	−0.52	Nucleus
OF29722-RA	*PbGLK24*	205	23,241.21	10.18	68.23	62.78	−0.989	Nucleus
OF04128-RA	*PbGLK25*	396	44,315.52	8.64	74.03	58.66	−0.851	Nucleus
OF23584-RA	*PbGLK26*	420	46,906.48	5.44	52.42	74.6	−0.658	Nucleus
OF14499-RA	*PbGLK27*	347	39,998.96	9.31	52.45	59.28	−1.022	Nucleus
OF02188-RA	*PbGLK28*	833	90,318.07	6.12	50.93	84.53	−0.268	Nucleus
OF03171-RA	*PbGLK29*	223	24,556.97	7.71	36.31	84.48	−0.481	Nucleus
OF09627-RA	*PbGLK30*	468	51,610.95	5.53	55.73	63.95	−0.746	Nucleus
OF29382-RA	*PbGLK31*	305	33,298.39	8.97	50.98	70.98	−0.755	Nucleus
OF11944-RA	*PbGLK32*	419	46,471.49	6.91	58.42	60.79	−0.741	Nucleus
OF21061-RA	*PbGLK33*	341	37,903.13	7.83	74.11	54.37	−0.945	Nucleus
OF00450-RA	*PbGLK34*	380	43,108.4	8.89	58.67	68.5	−0.782	Nucleus
OF06602-RA	*PbGLK35*	429	47,287.61	5.92	59.7	57.58	−0.879	Nucleus
OF11915-RA	*PbGLK36*	724	78,445.02	6.5	49.96	81.69	−0.31	Nucleus
OF28330-RA	*PbGLK37*	201	21,574.26	9.1	70.15	67.06	−0.602	Nucleus
OF15632-RA	*PbGLK38*	221	24,509.65	6.36	41.82	76.33	−0.524	Nucleus
OF09129-RA	*PbGLK39*	463	51,364.98	6.64	55.4	65.1	−0.794	Nucleus
OF23451-RA	*PbGLK40*	268	29,072.64	6.59	53.16	66.72	−0.504	Nucleus
OF20950-RA	*PbGLK41*	649	72,221.34	5.1	52.37	66.98	−0.565	Nucleus
OF14662-RA	*PbGLK42*	153	17,563.21	9.6	37.73	79.61	−0.644	Cytosol
OF09041-RA	*PbGLK43*	413	45,500.32	6.01	56.38	71.23	−0.616	Nucleus
OF02019-RA	*PbGLK44*	352	38,517.24	5.29	73.37	56.56	−0.716	Nucleus
OF02187-RA	*PbGLK45*	510	56,431.14	6.07	45.44	74.12	−0.487	Nucleus
OF02164-RA	*PbGLK46*	351	38,823.34	7.76	45.75	67.29	−0.605	Nucleus
OF18065-RA	*PbGLK47*	327	36,990.15	9.27	53.86	61.47	−0.823	Nucleus
OF23673-RA	*PbGLK48*	941	103,959.54	6.24	45.5	87.3	−0.273	Nucleus
OF25917-RA	*PbGLK49*	350	38,844.98	8.14	59.67	83.34	−0.635	Nucleus
OF11234-RA	*PbGLK50*	397	43,851.69	8.55	61.5	70.98	−0.675	Nucleus
OF15566-RA	*PbGLK51*	342	38,314.62	8.14	62.87	73.04	−0.696	Nucleus
OF27986-RA	*PbGLK52*	408	46,172.71	7.65	38.54	75.15	−0.554	Nucleus
OF11599-RA	*PbGLK53*	556	62,619.62	6.79	41.06	88.87	−0.354	Nucleus
OF09936-RA	*PbGLK54*	750	81,821.93	5.89	45.54	78.49	−0.409	Nucleus
OF06026-RA	*PbGLK55*	581	63,602.17	5.84	51.12	82.34	−0.306	Cytosol
OF11698-RA	*PbGLK56*	239	27,611.85	10.21	57.9	83.6	−0.657	Nucleus
OF23452-RA	*PbGLK57*	207	22,962.79	5.9	63.35	66.96	−0.757	Nucleus
OF23315-RA	*PbGLK58*	445	49,250.66	6.89	49.39	59.46	−0.747	Nucleus
OF19961-RA	*PbGLK59*	319	35,748.06	9.48	66.58	76.08	−0.688	Nucleus
OF22052-RA	*PbGLK60*	358	39,590.35	7.21	55.22	72.79	−0.659	Nucleus
OF23001-RA	*PbGLK61*	446	50,176.54	8.74	52.64	69.37	−0.666	Nucleus

^1^ aa: amino acid number; ^2^ MW: molecular weight; ^3^ pI: theoretical isoelectric point; ^4^ GRAVY: grand average of hydropathicity.

## Data Availability

Data is contained within the article and Supplementary Material.

## Supplementary Materials

The supporting information can be downloaded at: https://www.mdpi.com/article/10.3390/ijms26062387/s1.
